# Psychotherapy and Moralising Rhetoric in Galen’s Newly Discovered *Avoiding Distress (Peri Alypias)*

**DOI:** 10.1017/mdh.2014.54

**Published:** 2014-10

**Authors:** Sophia Xenophontos

**Affiliations:** Département d’ Histoire, Université Libre de Bruxelles, Arts et Archéologie, 50 Av. F. Roosevelt CP 175, 1050 Brussels, Belgium

**Keywords:** Galen, Graeco-Roman/Imperial period, *Peri alypias*, Distress, Psychotherapy, Practical ethics

## Abstract

In this article, I examine Galen’s credentials as an ethical philosopher on the basis of his recently discovered essay *Avoiding Distress* (*Peri alypias*). As compensation for the scholarly neglect from which Galen’s ethics suffers, I argue that his moral agenda is an essential part of his philosophical discourse, one that situates him firmly within the tradition of practical ethics of the Roman period. Galen’s engagement with Stoic psychotherapy and the Platonic-Aristotelian educational model affirms his ethical authority; on the other hand, his distinctive moralising features such as the autobiographical perspective of his narrative and the intimacy between author and addressee render his *Avoiding Distress* exceptional among other essays, Greek or Latin, treating anxiety. Additionally, I show that Galen’s self-projection as a therapist of the emotions corresponds to his role as a practising physician, especially as regards the construction of authority, the efficacy of his therapy and the importance of personal experience as attested in his medical accounts. Finally, the diligence with which Galen retextures his moral advice in his *On the Affections and Errors of the Soul* – a work of different nature and intent in relation to *Avoiding Distress* – is a testimony to the dynamics of his ethics and more widely to his philosophical medicine.

The philosopher’s lecture room is a ‘hospital’: you ought not to walk out of it in a state of pleasure, but in pain; for you are not in good condition when you arrive. Epictetus, *Discourses* 3.23.30

## 1. Some Preliminaries

Galen of Pergamum (AD 129–*c*.216), a central figure in the history of medicine and the intellectual culture of the Imperial period, has been at the heart of the scholarly activity over the last decade. The vibrant interest in him lies in his contributions to specialised fields of the medical art – from anatomy to physiology and dietetics to pharmacology – which were to influence Byzantine, Islamic, and Western medicine in significant ways. What is often less discussed is his affinity to philosophy, manifested *inter alia* in his profound philosophical education, his dialogue with prominent thinkers of the past (including Plato and Aristotle), and his self-perception as a philosopher-cum-physician.^1^ Recently there has been a tendency to explore Galen’s logic, epistemology, and natural philosophy in an attempt to show how these philosophical branches inform his scientific theory and practice.^2^ The same seems to be the case with the nascent work on Galen’s psychology, which provides us with the doctrinal theorisations concerning the structure, essence and function of the soul, but still does little to associate this descriptive model to its normative counterpart, ethics.^3^ Galen’s moral programme on how we should conduct our lives has been largely passed over or at best treated cursorily.^4^

## 2. Galenic Ethics and *Peri Alypias*

Galen exhibits a peculiar concern for ethics. In his auto-bibliographical work *On My Own Books*, he distinguishes a special category of twenty-three texts dedicated to moral philosophy (περὶ τῶν τῆς ἠθικῆς φιλοσοφίας ἐζητημένων).^5^ Of these three have come down to us, *On the Affections and Errors of the Soul* (in Greek), *Character Traits* (in Arabic summary), and the long-lost *Avoiding Distress* (in Greek).^6^ The latter is preserved in the Vlatadon 14 (fol. 10v–14v), a fifteenth-century manuscript from Constantinople, which was discovered by Antoine Pietrobelli in 2005 in the homonymous monastery in Thessaloniki.^7^ This has been considered an important work for several reasons: it offers valuable information about the production and publication of ancient books, and the holdings of Imperial libraries;^8^ it elucidates aspects of Galen’s life which we can cross-check against the unreliability of his Arabic biographies;^9^ finally, it adds to our knowledge of the regime of Commodus (AD 180–92), one of the cruellest Roman rulers. More than that, *Avoiding Distress* is, to my mind, a unique source for Galen’s identity as an ethical adviser, and, relatedly, for his didactic relationship with his audience, both direct and implied.

By focusing on the *Avoiding Distress*, its content, internal structure and narrative setting, I shall bring out the distinctive characteristics of Galenic ethics and evaluate its operation and impact within the framework of contemporary society. Some of the key topics with which I will deal are Galen’s departure from other moralists who have treated the issue of distress such as Seneca, Epictetus and Plutarch, and the extent to which Galen’s psychotherapy is informed by the (rhetorical) techniques he applies in his medical accounts, directed at the treatment of the body. Moreover, given that Galen’s *Avoiding Distress* is the only extant work *Peri alypias*,^10^ it may help us to shape an idea about the potential content of other lost essays on this topic.

## 3. *Avoiding Distress*: Generic Observations and Individual Features

*Avoiding Distress* is written as an essay-letter in response of a request by an anonymous friend of Galen. The correspondent wants to find out the philosophical mechanisms that ensured Galen’s self-control in the face of the great conflagration of the Palatine hill in AD 192. The chronology of the treatise makes part of the point, because its date in the early months of AD 193 shows how the reminiscence of the disaster would have still been a fresh memory – a revived reality, as I shall argue – in the minds of both author and addressee.^11^

The thematic framework makes it clear from the outset that this is an essay with moralising intent, belonging more generally to the popularised genre of practical ethics, a familiar genre in post-Hellenistic philosophical production, intended to have direct application in the lives of a broader class of reader.^12^ In referring to more specific typological distinctions among works of ethical philosophy, Philo of Larissa (158 BC–84 BC), once head of the Platonic Academy, presented a threefold categorisation: protreptic works (guiding towards morally adept courses of behaviour), therapy (applying philosophical guidance to particular cases of the treatment of emotions), and advice (proposing life-styles through which to achieve happiness by means of the therapy that has already been applied).^13^ It is in the ‘therapy of emotions’ that *Avoiding Distress* best fits, without excluding the generic interpenetration with the categories of the protreptic and the advice, issues to which I will return in the main section of this article. On the other hand, the essay’s prescription towards freedom from grief had led some critics to associate it with the genre of the consolation,^14^ from which, however, *Avoiding Distress* differs in certain respects: firstly, it does not involve the loss of a beloved person or (less often) one’s exile as the causes for grief, but rather material deprivation; secondly, it is not addressed to the person who mourns the loss, but to a philosophically minded addressee who seeks remedies for regaining equanimity in case of need.^15^

What renders *Avoiding Distress* rare among mainstream works of practical ethics is that the moral instruction professed throughout is enhanced with autobiographical touches, which contribute to a lively sort of moralisation. The therapy that is on offer, visualised through a very personal lens, helps to consolidate Galen’s role as a practical moralist, because it ensures that his prescriptions are a piece of counsel already tested and proved successful in the author’s moral experience. On another level, Galen’s tranquillity as opposed to the expected feeling of perturbation puts him in position to manipulate his readers’ emotional responses during the process of reading, as we shall see.

Since *Avoiding Distress* is a relatively unfamiliar text, it might be helpful to divide it into thematic units corresponding to the stages of Galen’s moralising argumentation (see Table 1).

**Table 1:** *Avoiding Distress*: thematic units corresponding to the stages of Galen’s moralising argumentation

1.	Introductory frame; Galen builds up his authority	§1–3, 66–67 KS  §1–8, 2–4 BJP
2.	He reconstructs the reality of the loss and stimulates the feeling of grief in his reader(s)	§4–16, 67–73 KS  §10–37, 4–13 BJP
3.	Transitory paragraph; Galen redirects the demands of the addressee and launches the therapy of grief	§16, 73 KS  §38, 13–§39, 13, 11 BJP
4.	In the course of the therapy, he engages with the major philosophical schools via tradition and innovation alike	§17–26, 73–79 KS  §39, 13, 12–§68, 21, 10 BJP
5.	His ethical advice is disseminated to a wider audience; conclusion of the essay	§27–31, 79–81 KS  §69–84, 21–26 BJP

## The Construction of Authority in the Preface: §1–3, 66–67 KS  §1–8, 2–4 BJP

4.

In the preface of epistolary tracts, the author traditionally mentions the incentive that had led to the composition of his literary work. Galen claims that he has received his correspondent’s letter, with which he requested information regarding the kind of training, arguments and considerations that made Galen immune to distress. Beyond this brief trope, it is striking how in the rest of the preface Galen reproduces the content of his friend’s letter by disclosing some of its main points in particular.

According to the narrator, the friend had been personally present and had observed (ἐωρακέναι, §1, 66, 3 KS  §1, 2, 6 BJP; cf. ἐωρακέναι, §1, 66, 7 KS = §2, 2, 11 BJP) Galen’s tranquillity after the latter was deprived of his slaves in the Antonine plague, while he was informed through oral reception (ἀκηκοέναι, §1, 66, 5 KS  §1, 2, 8–9 BJP) that Galen had suffered from similar setbacks in the past. The narrator additionally tells us that his correspondent now has a clear estimation of the losses of the fire (αὐτὸς ἔφης ἐπίστασθαι, §2, 66, 11 KS  §3, 2, 16 BJP), and that an informant had reported to him (πεπύσθαι δὲ τινος ἀγγείλαντός σοι, §2, 66, 11–12 KS  §3, 2, 16–3, 1 BJP) how Galen was not grieved, but continued cheerfully his normal activities.

The verbal forms of observation and cognition are used to reinforce the credibility of Galen’s account. His equanimity in response to a range of distressful events is backed up by external evidence, by people who have personally encountered him and now provide us with objective reports. Galen’s management of grief attracts the attention of those around him and leads progressively to his establishment as a moral exemplar. The origins of that are found in Galen’s detailed numeration of his losses, given in an ascending order from relatively minimal to more substantial, stressing thus the importance of the deprivation: many gold, silver plates, and loan documents, but also his drugs, both simple and compound, as well as his medical instruments; then, the editions of ancient authors he had prepared, his own compositions, and a rare collection of antidotes – among which the famous ‘theriac’, by far the most significant in the whole of the Roman world (§2, 66, 13–67, 28 KS  §4–6, 3, 3–4, 5 BJP). By confronting the destruction of those treasures with lack of distress, Galen stimulates his correspondent’s amazement (θαυμάζειν, §2, 66, 13 KS  §4, 3, 3 BJP). This is a topic with salient ramifications, because it now testifies to Galen’s self-projection as an ethical paradigm, juxtaposed to the counter-example of Callistus the grammarian (about whom the correspondent had also been informed, πεπύσθαι, §3, 67, 29 KS  §7, 4, 6 BJP): facing the loss of his books in the same fire, Callistus died of depression, surrounded by mourning people in black clothing (§3, 67, 29–32 KS  §7, 4, 6–10 BJP).^16^ The scene of death itself is contrasted to the cheerful disposition with which Galen withstood the losses mentioned above (φαιδρόν, §2, 66, 12 KS  §3, 3, 1–2 BJP), and ultimately lends legitimacy to his suitability to write a topic on the treatment of distress.

Another key issue arising from the preface is that of the role in the text of the addressee, who is meant to participate not just as a witness to the loss of Galen’s material goods, but most significantly by means of his personal rapport with the author. In reconstructing the addressee’s letter, Galen offers a clear glimpse of how the two men share common reminiscences and thus explains that their epistolary communication advances the exchange of knowledge and ideas. Later on in the text the proximity of the two men is reflected in Galen’s description of the social credentials of his friend, which so much resemble his own; he is a fellow Pergamene, of the same age as him (now in their sixties or early seventies), they have known each other from childhood, attended school together, and enjoyed the same liberal education (ἐξ ἀρχῆς συναναστραφεὶς καὶ συμπαιδευθεὶς, §21, 76, 243–245 KS  §51, 16, 18–19 BJP; σὺ παιδευθεὶς σὺν ἡμῖν, §24, 77, 271–272  §57, 18, 19–20 BJP). Having spent some time in Rome, the friend embraces Galen’s political perspective on Commodus’s politics (§22, 76, 257–261 KS  §54, 18, 1–6 BJP); and although they are now miles apart they have maintained a close friendship for many years. The recollection therefore of Galen’s personal misfortune is expected to be a familiar matter to the addressee, so that it makes the quest for ethical equilibrium a joint task as well. Galen is not a distant preacher, but an intimate ethical advisor.

The deliberate introduction of personality into the narrative might be explained in the light of what Galen suggests in his *Character Traits*: the aim of the good man is reforming his own soul to reform the souls of all other people over whom he has influence, one by one, beginning with the closest to himself – this he will achieve by teaching them by precept what they ought to do and by making himself an example to them in what he does.^17^ The general pattern brings to mind Michel Foucault’s view of how the care of the Self (in Galen’s case autobiographical introspection) can become a means to help the Other, and how a preoccupation with the particularity of the Other, his status as a person long known and dear to the author, facilitates the ethical treatment.^18^

On another level, Galen’s self-presentation as an ethical authority brings him close to the spirit of his self-projection in his medical case histories. These embedded clinical narratives, far more than exploring the stages of the diagnosis, treatment and prognosis of the diseases of particular patients, attest to Galen’s superiority as a physician, reanimating through auto-recollection the responses of his peers at his medical performances.^19^ The commonest of these responses is amazement.^20^ In one of Galen’s most fascinating writings *On Prognosis*, Eudemus admires Galen and advertises his medical competence to high-ranking personalities in Rome,^21^ while elsewhere Galen receives the admiration of the Imperial circle for curing the young emperor.^22^ Similarly, Galen’s medical efficacy is backed up by the addressee’s own confirmation of Galen’s claims (the frequent asides ‘as you very well know’),^23^ for he has been constantly present during the performances. Finally, although the author is presented as an exceptional physician, he is never isolated from his social circle (comprising a gamut of teachers, patients, and physicians). The communal experiences that he shares with his addressee in particular, and the direct interaction between the two expressed in the use of the sociative ‘we’ make Galen’s medical narration a social rather than a private act.^24^ It is this same tone that informs the *Avoiding Distress* and which suggests that in transmitting his personal ethical assertions, Galen is not alone but at the very core of his surroundings, a philosopher embedded within society – a point to which I will return.

When Galen was composing his *Avoiding Distress*, essays on psychic tranquillity were already in circulation, for instance, by Democritus and Panaetius (now lost), and by Seneca and Plutarch, philosophers with whom Galen enters into debate. Plutarch’s preface to his *On the Tranquillity of the Soul* in particular could work as a good *comparandum* regarding the issues of the construction of authority and the relation between author and addressee in such moralising sub-texts. This is an epistolary essay too, which Plutarch addresses to his Roman friend Paccius upon the latter’s request for an account on emotional resilience. In this case, Plutarch bases his philosophical potential not on his ethical experiences, which are totally absent from the essay, but on the philosophical material that he is able to elaborate in written form: a work of practical ethics after direct consultation of his personal note-books (*hypomnemata*) on the one hand^25^ and an exegetical commentary elucidating some difficult passages from Plato’s *Timaeus* on the other (464 E–F).^26^ In the rest of the essay, Plutarch is lecturing, seeking to direct his addressee’s conduct; for instance, he praises Paccius for not succumbing to the evils of fame and social standing (465A), and elsewhere castigates him on the suspicion of self-interest and overindulgence (468E). Still, as a character in the narrative, Paccius progressively fades away and becomes a constructed substitute for a larger audience that enjoys Plutarch’s protreptic moralism. The wider appeal of Plutarch’s essay is also attested by the fact that it is not context-specific (in the fashion of *Avoiding Distress*), but concerned with a large number of situations that may generate distress.

In Seneca’s *On the Tranquillity of the Soul* the author is also a qualified philosophical teacher, and his addressee a passive subject that needs to follow Seneca’s chain of precepts. Epictetus is a similar case in point, because his *Discourses* and *Manual* communicate to his fragile young students his ethical teachings through imperatives and hortatory subjunctives.^27^ Even the preface of Galen’s *On the Affections and Errors of the Soul* moves in the realm of such protreptic works of ethical philosophy, given that within a less intimate framework Galen encourages his reader towards certain philosophical positions, and any kind of shared memory between author and addressee does not seem to be in play. Galen’s *Avoiding Distress* represents an original use of the construction of authorial identity, in that author and addressee develop a dynamic relationship, as I will now show in more detail.

## The Revived Reality of the Loss: §4–16, 67–73 KS  §10–37, 4–13 BJP

5.

We have seen in the previous section that Galen refers to Callistus the grammarian, who died of distress at the loss of his books. Apart from functioning as a counter-intuitive paradigm, this incident helps to re-perform the public response to the fire. Galen reports that the majority of the people stored their possessions in the Temple of Peace, driven by the confidence (expressed with the recurrent cognates of θαρρεῖν, §3, 67, 33, 35, 40 KS  §8, 4, 1–11, 14, 21–22 BJP) that the repositories could not be damaged by fire. The tragic overturning of the expectations gives rise to their disappointment, from which Galen abstains.

In a new section he stresses that apart from the general disaster, he alone had to suffer a personal one ( ἴδιον, §4, 67, 41  §10, 4, 22 BJP) that made his misfortune more discomfiting: as he was about to visit his estate in Campania, he decided to store all his possessions in the repositories to make sure he kept them safe, but instead had everything destroyed. He was nonetheless not disturbed even for a moment, and this has motivated the addressee’s wish to request a first-hand account of the incident, although he has already learnt about it through witnesses, as seen. With his emotional separation from common reactions and his addressee’s acknowledgment of his exceptionality, Galen provides his reader with a sense of security; and as the narrative progresses he reconstructs a climactic description of the loss (more extensive and systematic than the one we have noticed in the preface), meant to generate a feeling of retrospective distress upon the reader. The author reforms his addressee’s behaviour by assigning him specific thoughts and corresponding emotional reactions. These manipulating strategies, as I call them, take the form of direct asides, given in the second-person singular, and verge on what we nowadays call the power of ‘suggestion’, a term coined by nineteenth-century psychologists such as William James.^28^

The asides start to appear in the juncture where we pass from the past tense, in which the correspondent’s epistle was reported, to the present tense, in which Galen now focuses on the after-effects of the loss. He says bluntly that even today he can feel the bereavement of everything essential for his practice every time he needs a book, instrument or drug (μέχρι νῦν αἰσθάνομαι καθ᾿ ἑκάστην ἡμέραν, §4, 68, 53 KS  BJP); and directly ‘suggests’ the first thought to his friend: ‘But the worst thing of all (δεινότατον) in the loss of my books has escaped your notice (λέληθέ σε): there is no hope (μηδὲ ἐλπίδα) of recovery, because all libraries on the Palatine were burnt on that same day’ (§4, 68, 54–57 KS  §11, 5, 12–15 BJP). In the reproduction of the friend’s letter, Galen allowed some hope in the recovery of his medical instruments, although he was clear that this demanded a significant amount of time and effort (§2, 67, 21–22 KS  §5, 3, 13–14 BJP), whereas here the elimination of any hope transposes a sense of retrospective despair upon the addressee:

For there is no possibility of finding either rare books that are not available elsewhere, or copies of common works which were excellent as regards the precision of their text, such as those of Callinus, Atticus, Peducaeus and even Aristarchus, including the two Homers, and the Plato of Panaetius, and many others of equal value . . .There were also autograph texts of many ancient grammarians, orators, doctors and philosophers (sc. that are now lost). (§5, 68, 58–65 KS  §13, 5, 18–6, 7 BJP)

The valuable legacy to posterity has been burnt to ashes, but Galen’s narration becomes more forceful when he claims that in addition to the numerous books, he also lost on that same day his recent editions, which were so carefully arranged so that ‘not even a marker, single or double, or a mark of crasis (ie. *coronis*) (was) displaced between books’ (§6, 68–69, 66–72  §14, 6, 13–15 BJP). In that category belonged the works of Theophrastus, Aristotle, Eudemus, Clitomachus and Phaenias, and most of Chrysippus and of all the ancient doctors (§7, 69, 75–77 KS  §15, 6, 18–21 BJP).

The same pattern recurs; the reference to a group of perished intellectual treasures is accompanied by two manipulative asides, which now stir up not the idea but the emotion of grief itself: ‘You will be particularly distressed to learn that (Λυπήσει δέ σε καὶ ταῦτα μάλιστα) I have found in the Palatine libraries some books not described in the so-called Catalogues’, some spurious, and others with limited circulation (§7–8, 69–70, 77–100 KS  §16, 6, 21–19, 8, 11 BJP). In similar fashion: ‘You would perhaps find particularly distressing ( ἴσως δὲ ἐλύπει  σε ἂν) the fate of my work on words in Attic Greek and everyday language’, comprising two parts, one drawn from old comedy and the other from prose-writers (§9, 70, 101–103 KS  §20, 8, 11–15 BJP).

In order to boost the reader’s anxiety through variation, Galen now adduces the role of fate (*tyche*), a traditional motive in ethical works of the Hellenistic period onwards, which is foregrounded also in his *Exhortation to the Study of Medicine*.^29^ Fate is a *media vox*, known at some times for its benevolence and at others for its unexpected blows that torment human life, and hence it shows the need for philosophical instruction as a preventive medium. Galen, so he tells us, had prepared copies of all his works intended for distribution, but had managed by chance (κατὰ τύχην, §9, 70, 103–104 KS  §20, 8, 15 BJP; cf. κατὰ τὴν τύχην, §12, 71, 141–142 KS  §28, 10, 21 BJP) to transport only the work on prose-writers to Campania, which is now saved. His remark that the same fate that favoured him also ambushed him (ἐνήδρευσεν . . . ἡ τύχη, §10, 70, 117 KS  §23b, 9, 11–12 BJP) is a step forward to the emotional climax, and leads Galen to dwell on the loss of his study of the vocabulary of old comedy, the significance of which he explains in no less than two paragraphs (§11–12, 71 KS  §24b, 9, 12–28, 10, 24 BJP).

As a contrast to the feeling of distress with which the addressee is now afflicted, Galen in turning to his own emotional state insists that none of these losses grieved him (τούτων οὖν οὐδὲν ἠνίασέ με, §13, 71, 144 KS  §29, 10, 24–25 BJP; ἀλλ᾿ οὐδὲ ταῦτα ἐλύπησεν, §13, 72, 149 KS  §30, 11, 7 BJP) although they were substantial and hard to replace, not even the destruction of his *hypomnemata* and a large number of medical and philosophical works. The emotional gap between the two parties turns the primary as much as the secondary audience to utmost wonder: ‘What then, you will say, is there anything more important than all that I have just described that could cause distress? Well, I shall tell you.’ (§13, 72, 149–151 KS  §31, 11, 7–9 BJP). Galen was convinced that he possessed the most remarkable drug recipes in the Roman empire, brought to him by a twofold kind of fate (Διττή ... τύχη, §14, 72, 154 KS  §32, 11, 13 BJP). His hypothetical question, however, and especially his assertive remark ‘I will tell you’ at the end are misleading, as we do not really get any answer on whether the loss of his drugs upset him or not. Instead, Galen redirects the demands ascribed to his addressee, who does not care any more about which of the many destructions could have aroused more distress to Galen (obviously none), but how he was not grieved like other men at the loss of such a great variety of possessions (§16, 73, 181–184 KS  §38, 13, 3–8 BJP). The text is by itself a suggestive entity, conveying to the reader the idea that the authorial self is a unique role model among his contemporaries, whereas he would himself be one of those ‘others’ troubled by distress. Galen has helped the reader develop an introspective consciousness of his psychic frailty and realise the pressing need for its therapy.

## Traditional Instruction and Galen’s Ethics: §17–26, 73–79 KS  §39, 13, 12–§68, 21, 10 BJP

6.

In this section of the essay, Galen exploits the repertory of ethical instruction, familiar from the works of other moralists. One notices, for instance, the use of moralising anecdotes and quotations from authorities, to which Galen adds his individual twists. His place in the legacy of practical ethics is confirmed by the reminder he puts in the mouth of his addressee that the latter has heard him expounding similar pronouncements many times in the past (§16, 73, 185–187 KS  §39, 13, 9–11 BJP).

The moralising part starts with Aristippus of Cyrene, an important follower of Socrates who became proverbially known in ethical literature for his worldly hedonism. Aristippus appears also in Plutarch’s *Tranquillity of the Soul* 469C-D, where he is an example of a wise man rising above the unpleasant conditions of life.^30^ In contrast to Plutarch, Galen recounts two anecdotes about Aristippus, which point to the importance of self-sufficiency and hence to the idea that the loss of wealth should not be a matter of grief. Furthermore, Galen intertwines the moral of Aristippus’ anecdote with his own ethical voice, when he declares that he shares Aristippus’ view:

he (sc. Aristippus) shows very neatly what you have many times heard me say, that one should not focus on what has been lost, but consider how those who have inherited three fields from their father will not stand looking at others with thirty. (§18, 74, 201–204 KS  §42, 14, 7–11 BJP)

Galen offers mind-controlling techniques that secure happiness: that we should refrain from having too many desires which can hardly be satisfied, and be content with what is sufficient to life, both stances akin to what we call ‘attitude of gratitude’ in modern psychology,^31^ and which go as back as Epicurus.^32^ His advice becomes more attractive, when he provides moral evaluation of what is considered ethically great (§18, 74, 216 KS  §45, 15, 6 BJP).^33^ To his mind, the noble person is not the one who is not distressed after he had been left with three fields, but the one who is destitute and still bears his poverty without distress, such as Crates, Diogenes, and Zeno, who is a reflection of self-sufficiency also in Plutarch’s account (467C-D).^34^

Galen has so far employed conventional material to present his moralising in a protreptic tone, but again a personal note enters into the discussion. He explains in two instances (§19, 75, 219–220 KS  §46, 15, 10–12 BJP and §20, 75, 231–232 KS  §49, 16, 3–4 BJP) that it was not a great thing for him to despise the loss of his possessions, because he was always left with much more than sufficient. In similar vein, it was neither important when he despised his place at the imperial court (§20, 75, 231–234 KS  §49, 16, 3–8 BJP), nor when he had lost all his drugs, all his books, the recipes of major drugs, and the writings on them he had prepared for publication along with many other treatises (§20, 75, 234–240 KS  §50a, 16, 8–15 BJP). The recapitulation of his losses in reverse order is here not simply a reminder, but an ethical strategy with more complex connotations. We have seen in a previous section that upon his return to Rome Galen used to realise every day the importance of the loss and constantly found himself in need of particular books, instruments, or drugs. All these he now considers superfluous, and his particular use of the participle καταφρονήσαντι (‘having despised’) echoes the Stoic belief of indifferents:^35^ the only thing that determines happiness is virtue and everything else, including wealth, health, fame and social prominence, are moral indifferents, factors that cannot affect individual happiness.^36^

Galen’s philosophical spirit is practical rather than theoretical, especially in instances such as these in which he talks as a social critic, aiming at correcting the deviant morals of those around him. Second-century Graeco-Roman society is often seen – and was seen at the time – as a deeply competitive one, in which personal elevation became a means in itself, very often ignoring the importance of morality. In the introduction of his *On Prognosis*, the two proems to the *Method of Healing*, and his *Recognising the Best Physician*, which take the form of ethical diatribes, Galen comments on the degeneracy of his times and castigates in particular the corruption that afflicted physicians in Rome.^37^ He frequently expresses his wish to abandon the capital and move back to his native town, which he paints in a more positive light. The spatial segregation between Rome and the Greek East must be a token of Galen’s Hellenism and refers to the topicality of this subject in what is named as the ‘Second sophistic’ period, especially when it came to the responses of Greek intellectuals under Roman rule.^38^ We will see below that Galen criticises Commodus’s rule, and we have already observed that Galen’s addressee is a fellow Pergamene and not a Roman dignitary, like Plutarch’s Paccius.

On another level, it is important to note that in his public debate within *Avoiding Distress* designed to advise contemporary readers, Galen adopts some of the tenets of Stoicism (despite his general hostility to Stoic psychology), because this was considered a very fully worked out kind of philosophy and way of life, and hence one that suited his practical spirit. Galen, for instance, suggests the method of the *praemeditatio futurorum malorum*, one of the fundamentals of Stoic psychotherapy, though shared with the Cyrenaics, according to which the anticipation of negative experiences might lead to an increased ability to endure them when they come.^39^ Although this meditative practice seems to be a contested one among philosophers – Epicurus, for example, claimed that grief is either bearable or short and that we should thus not aggravate it by focusing on its imagined visualisation,^40^ Galen is openly in favour of it (§21, 76, 248–253 KS  §52, 17, 4–9 BJP).^41^ Again the moralising is not simply thrown at the addressee as a precept to adopt without further consideration, but it becomes an element in their common experiences. The addressee is actively involved in the narrative, when Galen reminds him of the crimes committed by Commodus and how these political fears schooled Galen’s imagination (ἐγύμνασά μου τὰς φαντασίας, §22, 76, 260–261 KS  §54, 18, 5–6 BJP) to prepare him for the total loss of all his possessions. The notion of φαντασία is again of Stoic parentage, referring to the impressions that are created in one’s mind upon the stimulation of one’s senses at external phenomena, and Galen advises his friend to practise his own imagination too (ἀσκεῖν παρακελεύομαι τὰς φαντασίας σου τῆς ψυχῆς, §24, 77, 268 KS  BJP) by expecting to be confronted with the likelihood of exile, an open threat under the regime of Commodus (§23, 89–90 KS  §56, 18, 15–16 BJP). Here Galen is certainly addressing a broader group of people who must have felt the capricious politics of the Roman emperor, and gives them practical ethical means to withstand possible dangers deriving from his oppression.

The practical tone of Galen’s ethics is not the product of his Stoic leanings or social commentary alone, but of his personal experience as well. Around the end of the essay Galen raises a central issue in ancient ethics, when he claims that his training of imagination was predicated upon a combination of proper natural propensities and excellent education (§24, 77, 269–272 KS  §57, 18, 17–20 BJP). This gives him the opportunity to discuss the contribution of his father to his ethical training, a topic of which Galen is very fond.^42^

Galen’s ethical agenda was indebted to the Platonic and Aristotelian educational model, which maintained that human character was shaped by the right mixture of nature (*physis*) and environmental training (*askesis*). Although in the passage we have just seen he brings out both aspects as informing his own education (also §25, 77, 282–284 KS  §60, 19, 10–13 BJP), in referring to his father he makes clear that he was a naturally endowed man, who possessed his ethical qualities without having been exposed to the influence of philosophers (§24, 77, 275–281 KS  §58, 19, 2–§58, 19, 2–§59, 19, 10 BJP). Galen’s position on *physis* is a very complex one, because he uses it with semantic flexibility across a great variety of texts. For instance, in his *Character Traits* he talks about features of character that appear in infants as soon as they are born, and, relatedly, states that anger and revenge are inherited traits in man, not learned.^43^ The significance of *physis* is lessened in the *Affections and Errors of the Soul*, where nature together with early education and rational inputs represent the educational triad that made Galen immune to distress;^44^ the same tone prevails in his *Natural Faculties*,^45^ whereas in his *magnum opus*, *The Doctrines of Hippocrates and Plato* training is set above nature.^46^ In *Avoiding Distress*, the triad excludes *physis* altogether and replaces it with rational arguments instead (§1, 66, 1–2  §1, 2, 1–2 BJP). The retexturing of the same notion should be explained in the light of the argumentation and its purposes each time.

Galen devotes a separate section of the *Affections and Errors of the Soul* to narrate the case of a young man in his close circle, who was surprised that Galen was not vulnerable to great disasters, while he himself was distressed even by trivial ones. In asking for Galen’s explanation for this, the latter replied that ‘nature has great power in childhood, so too does emulation of those amongst whom one lives, then at a later stage the important factors are doctrines and training’ (25, 21–24 DB  37 K). Textual evidence suggests that this essay is indeed addressed to a young man whose philosophical background is still an elementary one; for instance, he is in need of a moral supervisor to criticise his conduct (36, 16–17 DB  55 K),^47^ and there is an emphasis on the moral drawbacks especially congenial to young people (26, 12–14 DB  38 K),^48^ both ideas not present in *Avoiding Distress*. Therefore, the omission of *physis* as a determinant of psychic harmony at the beginning of *Avoiding Distress* fits the advanced philosophical stage of his addressee, who was expected to be indifferent to an aspect that affects the initial stages of one’s training. On the other hand, the focus on the natural background of Galen’s father would have no place in an essay meant to teach young men the importance of correct training, whereas its occurrence at a later point in *Avoiding Distress* might be a useful motivation for a person towards the end of his life to proceed to a retrospective evaluation of the role of *physis*, now that the effects of adolescence training are not any more in play. Finally, the insertion of *physis* in this context reflects Galen’s philosophical eclecticism, because it is characteristic of Platonic-Aristotelian educational thinking on ethical development, but not Stoic, according to which early influences and instruction alone shape the moral character of the man.^49^

Galen’s way of treating the issue of the therapy of distress in the two accounts casts light on his credentials as an ethical author, so I want to stay for a while on this comparison. Galen describes how his father’s indifference to worldly pleasures (§25, 77, 284–78, 301 KS  §61, 19, 13–§64, 20, 14 BJP) determined his own scorn for fame, wealth, and social standing (§26, 78, 302–304 KS  §65, 20, 14–17 BJP). In the *Affections and Errors of the Soul* Galen’s father again becomes a model for him, but in this instance clearly contrasted to the one of his mother:

I did have the great good fortune to have a father who was to an extraordinary degree free from wrath, just, good, and generous; and a mother whose irascibility was so great that she would sometimes bite her maids. She was constantly shouting and fighting with my father, even more so than Xanthippe with Socrates. Thus, as I saw in comparison the fine qualities of my father’s deeds and the ugly affections to which my mother was subject, I embraced and loved the former, and avoided and hated the latter. I observed a very great difference between my parents in this respect; and so too in the fact that my father never appeared distressed at any setback, while my mother would suffer grief at the smallest occurrence.^50^

The structure of the passage (with the polarised distinction between the good father and the bad mother) shows that the text is meant to appeal to an audience of philosophical novices. But more than that, it is interesting how in the *Affections and Errors of the Soul* Galen’s father teaches him, among other things, the avoidance of distress, which Galen refrains from mentioning in the corresponding part of the *Avoiding Distress* where it would have sat easily:

I had always recalled the counsel that my father gave me: that one should not be distressed by any material loss provided that what remains is adequate for the care of one’s body. This he laid down as the primary aim of possessions: to keep one from hunger, cold or thirst. If one happens to have more than is necessary for these purposes, one should, he believed, use it for good works. I have, indeed, up to now had access to sufficient resources to bestow in this way, too.^51^

The pedagogical thrust received from his father during his formative years in the *Affections and Errors of the Soul* has been internalised by Galen, and he now transmits it as a mature philosophical authority in his *Avoiding Distress*. The link between the two essays testifies to Galen’s consistent train of ethical thought; and the elements of variation according to the requirements of his text each time indicate the creativity with which he remodels the impact of emotions. In his proem to the second part of the *Method of Healing*, a technical work addressed to an experienced doctor, Eugenianus, Galen admits that he was distressed over a long period of time, so that he had been unable to touch a book.^52^ Although this remark is at odds with the suggested imperturbability one encounters in his ethical works, it is important that Galen makes no claims to be an exemplum of moral reflection in this work. Furthermore, his distress in the prefatory passage of the second part of this work could be a rhetorical explanation for the twenty-year gap of composition between the first section of the work (Books 1–6) and the second one (Books 7–14).^53^

## Philosophical Refutation via Personal Experience: §27–31, 79–81 KS  §69–84, 21–26 BJP

7.

In the last part of the treatise, Galen is directly involved with philosophical debates regarding distress, and in particular with the definition of *apatheia*. The passage is of assistance in seeing how he understands the notion and in making us aware of the way he deploys it with syncretic flexibility rather than sectarian devotion. Although there are instances where Galen is a proponent of the Aristotelian moderation of emotions, known as *metriopatheia* (‘hitting the mean’), there are other instances, for instance in his *Affections and Errors of the Soul*, in which he seems to advocate complete freedom from affection. In *Avoiding Distress* he rejects Stoic *apatheia*, as he hurries to make clear that he has never seen anyone so wise as to be entirely free from affections (§27, 79, 321–322 KS  §70, 21 §71, 21, 17–19 BJP). Here the allusion is to the Stoic sage, a paradigm of emotional imperviousness, with whom Galen does not want to be identified. In relation to this, the *apatheia* he has shown throughout the essay bears its limits:

I do not care about the loss of possessions up to the point in which I am not deprived of them all and sent to a desert island, (sc. I do not care about) bodily pain unless I am being placed in the bull of Phalaris. (§27, 79, 322–325 KS  §71, 21, 19–22, 2 BJP)

The bull of Phalaris is now an allusion to the Epicurean sage, who in light of his total *ataraxia* was expected to consider that life was pleasant even when suffering bodily tortures.^54^ Scholars have been perplexed by Galen’s blurred stance towards *apatheia* and *metriopatheia*,^55^ but at least in *Avoiding Distress* I think that Galen supports a modified version of *apatheia*, freedom not from all emotions but from violent and disruptive ones.

The regulated *apatheia* professed on a philosophical level also squares with Galen’s self-depiction as an ethical exemplar. His admission that the destruction of his homeland or a friend’s punishment by a tyrant could cause him distress (λυπήσει δέ με, §27, 79, 325–328 KS  §72a, 22, 2–4 BJP) shows that he now wants to be seen as an approachable model to his readers, since his previous self-control would have been beyond the capacity of normal people. So he goes on to pray to Zeus that no distressing event will ever happen to him. In similar vein, he accepts his human weaknesses and acknowledges the unexpected frustration that might occur from changes to his bodily and psychic state. Galen’s counsel against grief is here invested with his practical experience, because he accepts that he never claims to be able to do what he had not displayed in practice (§28, 80, 352–355 KS  §78b, 23, 14–24, 2 BJP). His experience in this instance is a vehicle of persuasion.

His ethical optimism is also seen in the final address to his correspondent, which places him alongside Galen in terms of nature and education (§29, 80, 364 KS  §79b, 24, 12–13 BJP) – a sign of merit for the addressee. Before the closure two categories of people are established, the former represented by Galen’s addressee who prefers simple food and dressing and is sexually restrained, the latter group includes all those people who are enslaved to sexual desires and can never satisfy their longing for money (§29, 81, 364–370 KS  §79b, 24, 13–25, 5 BJP). Galen’s ethics feeds into the realities of contemporary life, because it acknowledges the tendency for social and political prestige to drive people in upper circles. Here he connects patterns of behaviour to different types of people, and reinvigorates assimilation and distancing strategies:^56^ those people who are moderately attached to esteem, wealth, reputation and political power have only limited chances to be afflicted by distress; people whose desires for public reputation are insatiable will lead miserable lives, unaware of the virtue of the soul, and with toilsome grief (§30, 81, 371–378 KS  §81, 25, 6–§83, 25, 17 BJP).^57^ In assessing the two groups Galen also plays with his audience’s sense of honour, a pivotal quality of Graeco-Roman aristocracy.

On another level, this is a good example of how the essay has also features of the genre of advice (as distinct from that of therapy, according to Philo’s classification), since it proposes a particular life-style, which he hopes the reader will follow. Finally, the two kinds of ethical attitude do not stem from theory but from experience, which Galen now considers a teacher of the unexpected (᾿Αλλ᾿ ἡ πεῖρα καὶ τῶν ἀπροσδοκήτων διδάσκαλος γίνεται, §30, 81, 378 KS  §83, 25, 15–16 BJP). In Galenic ethics, experience is thus a means of premeditation and a guarantor of success, as it is indeed in technical contexts, for instance in his *The Capacities of Foodstuffs* ^58^ or in his pharmacological essays, *Composition of Medicines according to Types* and *Composition of Medicines according to Places*.^59^ At the same time, it is also a philosophical motivation for the composition of ethical works: at the end of *Avoiding Distress* Galen’s daily experience with ordinary men stimulates him to reflect on the topic of love of wealth (φιλοπλουτία) – a traditional one in the remit of practical ethics – and write a separate treatise on that, which he also sends to his friend (§31, 81 KS  §83, 25, 16–21–§84, 26, 1–3 BJP).

## 8. Concluding Remarks

In this article, I have explored Galen’s characteristics as an ethical philosopher in the light of his newly discovered *Avoiding Distress*. I have shown that his personal reflections on the issue of anxiety during a particular event from his life help him to build a strong ethical voice; and that by reviving his experiences as the victim of the calamity he convinces his reader(s) that he has firm knowledge of how to dispel grief at similar cases. Galen’s correspondent, who is not a mere literary construction but an active associate in the process of reading, reflects Galen’s intimate ethics, and on another level makes his psychological therapy more effective. The exposition of Galen’s losses is given climactically and is permeated with his manipulative asides, which suggest to his reader considerations and emotions that retrospectively enliven the feeling of distress until they ultimately free him from it. On many occasions, Galen’s applied ethics fits his self-sketching as a practising physician, especially as regards the construction of his authority, the credibility of his account, the importance of personal experience, and the issue of the amazement at his performances. All these peculiar features are hardly expected to occur in other lost *Peri alypias* essays, although that question must remain a speculative one.

In his *Avoiding Distress*, Galen resorts to moralising devices of considerable sophistication by combining philosophical remedies from different schools of thought; the rich Stoic background mixed with material from the Platonic and Aristotelian lines shows that he wants to situate himself firmly within this genre. His statement around the end of *Avoiding Distress* that he has written this essay neither with zealous enthusiasm nor considering it as an important task, but simply as a sort of hobby (§26, 78, 310–311 KS  §67, 21, 3–5 BJP) is nothing more than a trope of self-effacement. The impact of his ethics not only in the *Avoiding Distress* but also as it evolves in the *Affections and Errors of the Soul*, expressed in the subtle retexturing of the ethical instruction according to the philosophical level of his addressee and the needs of the argumentation each time, has proved to the contrary.

Galen’s moral essays are carefully crafted pieces of literature; and if we are to do justice to this output, we should refrain from regarding them as marginal, second-rate, works in comparison with his core (medical) writings, classifications that unavoidably bear evaluative connotations.^60^ Galen himself would have never drawn this kind of distinction, and his auto-bibliographical books, which provide numerous subgroups of his own medical and philosophical works, never reveal any preference for some over others. The scholarly view that Galen’s ethics cannot meet the standards of the philosophical language of his near-contemporary theorists^61^ is not consistent with the genuine twists of his ethical discourse, not least with the multiplicity of moral works circulating in this period, which was one that valued personal variation of ideas rather than uniformity and adherence to doctrinal authority.

But as the best judge of success is always the audience, at least as far as *Avoiding Distress* is concerned its programme of psychological therapy did not benefit only contemporary readers. The Vlatadon manuscript preserves several lines of Byzantine verse thanking Galen for his ethical precepts:

Thanks be to you, Galen, for your advice, in which you teach mortals to bear without grief the uncertainties of life, and not be disturbed at all by the losses; for at such a moment there is no creed, but in another’s misfortune, you are a clear beacon in your life – I do not say that you are a money-seeker,^62^ you will teach them very well how not to be bemused by chance events, even if you teach them through your own life.^63^^,^ 64

The Byzantine reception of the text encapsulates the main point I have made here, that the author’s life experiences profoundly inform the suggested cure for distress. Most significantly, it affirms Galen’s contribution as a philosopher whose intellectual ambitions embrace the therapy of the emotions on a larger social scale.

## Footnotes

1. By Galen’s own admission, the emperor was referring to him as ‘the first among doctors and unique among philosophers’, *On Prognosis*, 11, Vivian Nutton (ed.), *Galeni De Praecognitione*(Berlin: in aedibus Academiae litterarum, 1979), *CMG*, vol. V.8.1, 128, 27–28 /article/back/fn-group/fn/p/mixed-citation/inline-formula  Karl Gottlob Kühn (ed.), *Claudii Galeni Opera Omnia* (Leipzig: Carl Cnobloch, 1821–33), vol. 14, 660; elsewhere he claims that his teacher, the Peripatetic Eudemus, knew him for his philosophical standing, considering medicine to be a sideline for Galen, *On Prognosis* 11, Nutton (ed.), *Galeni De Praecognitione*, *CMG*, vol. V.8.1, 76, 27–29 /article/back/fn-group/fn/p/mixed-citation/inline-formula  Kühn (ed.), *Claudii Galeni Opera Omnia*, vol. 14, 608.

2. A few indicative examples will suffice; logic: Jacques Jouanna, ‘Does Galen have a medical programme for intellectuals and the faculties of the intellect?’, in C. Gill, T. Whitmarsh, J. Wilkins (eds), *Galen and the World of Knowledge*(Cambridge: Cambridge University Press, 2009), 190–205; epistemology: Robert James Hankinson, ‘Galen on the limitations of knowledge’, in Gill *et al.*, *Galen and the World of Knowledge*, 206–42; natural philosophy and physiology: Rudolph Siegel, *Galen’s System of Physiology and Medicine* (Basle: Karger, 1968), Robert James Hankinson, ‘Body and soul in Galen’, in R.A.H. King (ed.), *Common to the Body and Soul: Philosophical Approaches to explaining Living Behaviour in Greco-Roman Antiquity* (Berlin: Walter de Gruyter, 2006), 231–58.

3. The major representative is Christopher Gill: ‘Did Galen understand Platonic and Stoic thought on the emotions?’, in J. Sihvola and T. Engberg-Pederson (eds), *The Emotions in Hellenistic Philosophy*(London: Kluwer, 1998), 113–48, and *Naturalistic Psychology in Galen and Stoicism* (Oxford: Oxford University Press, 2010), mainly 243–329; also Robert James Hankinson, ‘Galen’s Anatomy of the Soul’, *Phronesis*, 36, 3 (1991), 197–233, and ‘Actions and passions: affection, emotion and moral self-management in Galen’s philosophical psychology’, in J. Brunschwig and M.C. Nussbaum (eds), *Passions and Perceptions: Studies in Hellenistic Philosophy of Mind* (Cambridge: Cambridge University Press, 1993), 184–222; Teun Tieleman, ‘Galen’s Psychology’, in J. Barnes and J. Jouanna (eds), *Galien et la philosophie*: *Huit exposés suivis de discussions* (Geneva: Fondation Hardt, 2003), 131–69; Pier Luigi Donini, ‘Psychology’, in R.J. Hankinson (ed.), *The Cambridge Companion to Galen* (Cambridge: Cambridge University Press, 2008), 184–209. See also the chapters in the volume by P. Manuli and M. Vegetti (eds), *Le Opere Psicologiche di Galeno* (Naples: Bibliopolis, 1998).

4. Scholars have only discussed Galen’s medical ethics in the essay *That the Best Doctor Is also a Philosopher*in relation to Hippocrates; Jacques Jouanna, ‘Galen’s reading of Hippocratic ethics’, in J. Jouanna (ed.), *Greek Medicine from Hippocrates to Galen: Selected Papers*(Leiden: Brill, 2012), 259–85.

5. 15, Veronique Boudon-Millot (ed.), *Galien*, *Œ**uvres, Tome I: Introduction générale; sur l’ordre de ses propres livres; Sur ses propres livres; Que l’excellent médecin est aussi philosophe*(Paris: Belles Lettres, 2007), 169, 13 /article/back/fn-group/fn/p/mixed-citation/inline-formula  Kühn (ed.), *op. cit.* (note 1), vol. 19, 45. Most of Galen’s works on moral philosophy bear titles that are traditional in the essay writing on practical ethics: eg. *Attendance at Dialogues*, *Pleasure and Pain*, *On Modesty*, *On Slander*, *Things Said in Public against Flatterers*, *On Encouragement*

6. The earliest mention to *Avoiding Distress* is in a ninth-century catalogue of Galen’s works provided by Hunain ibn Ishāq in his *Risāla*; see G. Bergsträsser (ed.), *Hunain ibn Ishāq: Über die syrischen und arabischen Galen-Übersetzungen*: *Abhandlungen für die Kunde des Morgenlandes 17.2* (Leipzig: Brockhaus, 1925), 40, no. 120. We know that it was translated into both Syriac and Arabic, although none of the translations survive today. In the late 12th and early 13th centuries, Joseph Ibn Aknı̄n, student of Maimonides, quoted many passages from *Avoiding Distress* in his Arabic *Hygiene of the Soul*; Abraham S. Halkin, ‘Classical and Arabic Material in Ibn Aḳnı̄n’s “Hygiene of the Soul”’, *Proceedings of the American Academy for Jewish Research*, 14 (1944), 25–147: 60–147. Afterwards it was cited by other Arabic and Hebrew authors of the 13th century; see Mauro Zonta, *Un interprete ebreo della filosofia di Galeno: gli scritti filosofici di Galeno nell’opera di Shem Tob ibn Falaquera*(Turin: S. Zamorani, 1995), 113–23 and BJP, LXX–LXXIV for additional information.

7. For a description of the manuscript, see Antoine Pietrobelli, ‘Variation autour du *Thessalonicensis Vlatadon* 14: un manuscrit copié au xénon du Kral, peu avant la chute de Constantinople’, *Revue des études byzantines*, 68 (2010), 95–126. The text was first published by Veronique Boudon-Millot in ‘Un traité perdu de Galien miraculeusement retrouvé, le *Sur l’inutilité de se chagriner*: Texte grec et traduction française’, in V. Boudon-Millot, A. Guardasole and C. Magdelaine (eds), *La Science médicale antique: Nouveaux regards: Études réunies en l’honneur de Jacques Jouanna*(Paris: Beauchesne, 2007), 73–123 (henceforth BM). A rev. edn followed: V. Boudon-Millot, J. Jouanna and A. Pietrobelli (eds), *Galien, Tome IV: Ne pas se chagriner* (Paris: Belles Lettres, 2010) with French translation and commentary (henceforth BJP). The references I give to the Greek text are primarily based on the edition of P. Kotzia and P. Soteroudis of the same year, P. Kotzia and P. Sotiroudis (eds), ‘Γαληνοῦ Περὶ ἀλυπίας᾿’, ῾Ελληνικά, 60 (2010), 63–148 (henceforth KS), with cross-references to the BJP edition for the reader’s convenience (note that the two edns present different chapter division and lining). It is striking that the text of Kotzia and Sotiroudis (otherwise easily available in all major libraries) has been neglected by modern scholars, despite the fact that they were the only editors who had autopsy of the manuscript in preparing their edition. This assisted them on palaeographical grounds to reconstruct corrupted words which the other editors have not been able to identify with certainty. For instance, in §4, 68, 49 KS /article/back/fn-group/fn/p/mixed-citation/inline-formula  §11, 5, 8 BJP, Kotzia and Sotiroudes read α– at the beginning of the word, and –τα at the end of it, and hence supplemented three missing letters in the middle –παν–, suggesting ἄ[παν]τα, whereas the French editors only read α– as the initial letter of a word, suggesting α[ὐτὰ]. Similarly, §4, 68, 51 KS suggest ἐπ[ανῆλθον], but §11, 5, 10 BJP cannot possibly supplement the word, since a beginning epsilon (ε–) is the only letter they can read. Additional reasons that led me to prefer the KS edn is their rich apparatus of parallel passages in contrast to the meagre one of BJP, and their detailed commentary, which provides extensive remarks on philological, philosophical and medical matters arising from the text. Further emendations on corrupted passages have been suggested by Ivan Garofalo, ‘Congetture inedite’, *Galenos*, 2 (2008), 137–8; Ioannis Polemis, ‘Διορθωτικά στο Περί αλυπίας του Γαληνού᾿, Επιστημονική Επετηρίδα της Φιλοσοφικής Σχολής του Πανεπιστημίου Αθηνών, 43 (2011), 1–8; Antonio Stramaglia, ‘Libri perduti per sempre: Galeno, “De indolentia” 13; 16; 17–19’, *Rivista di Filologia e di Istruzione Classica*, 139 (2011), 118–47; Anargyros Anastassiou, ‘Galen, de indolentia, §71, S. 21, 17–19 Boudon-Millot, Jouanna /article/back/fn-group/fn/p/mixed-citation/inline-formula  §27, S. 79, 321–322 Kotzia, Sotiroudis /article/back/fn-group/fn/p/mixed-citation/inline-formula  §71, S. 44 Garofalo, Lami, S.’, *Galenos*, 6 (2012), 49–51; Ivan Garofalo and Alessandro Lami, *Galeno: l’anima e il dolore: de indolentia, de propriis placitis* (Milan: BUR, 2012), 149–55. The translations of *Avoiding Distress* cited throughout are mine; the translations of Galen’s other ethical works are appropriated versions of the translations contained in Peter Singer (ed.), *Galen: Psychological Writings: Avoiding Distress, Character Traits, The Diagnosis and Treatment of the Affections and Errors Peculiar to Each Person’s Soul, The Capacities of the Soul depend on the Mixtures of the Body* (Cambridge: Cambridge University Press, 2014). In the same volume there is a new English translation of *Avoiding Distress* by V. Nutton, 77–99.

8. Pier Tucci, ‘Galen’s Storeroom, Rome’s Libraries, and the Fire of AD 192’, *Journal of Roman Archaeology*, 21 (2008), 133–49; Christopher Jones, ‘Books and Libraries in a Newly Discovered Treatise of Galen’, *Journal of Roman Archaeology*, 22 (2009), 390–8; Vivian Nutton, ‘Galen’s library’, in Gill *et al.*(eds), *op. cit.* (note 2), 19–34; Amneris Roselli, ‘Libri e Bibliotheche a Roma al Tempo di Galeno: la Testimonianza del *De Indolentia*’, *Galenos*, 4 (2010), 127–48; Matthew Nicholls, ‘Galen and Libraries in the *Peri Alupias*’, *Journal of Roman Studies*, 101 (2011), 123–42; Clare Rothschild and Trevor Thompson, ‘Galen’s On the Avoidance of Grief: The Question of a Library at Antium’, *Classical Philology*, 107, 2 (2012), 131–45.

9. SimonSwain, ‘Beyond the limits of Greek biography: Galen from Alexandria to the Arabs’, in B. McGing and J. Mossman (eds), The Limits of Ancient Biography (Swansea: Classical Press of Wales, 2006), 395–427

10. Eratosthenes of Cyrene (third century BC), Diogenes of Babylon (2nd c. BC), and Plutarch (‘The Catalogue of Lamprias’ no. 172) were all said to have written a lost essay *Peri alypias* I came across a work by John Klimax of the 7th c. AD, with the title Περὶ ἀπροσπαθείας, ἤγουν ἀλυπίας (*On Tranquility of the Soul, or rather on Avoiding Distress*, Book 2 from his *The Heavenly Ladder*), an interesting example of how Stoic moral notions were appropriated into the context of Christian ethics.

11. For the date of the essay’s composition, see Boudon-Millot, ‘Un traité perdu’, *op. cit* (note 7) 76, BJP,LVIII–LIX.

12. ParaskeviKotzia, ‘Galen, Περὶ ἀλυπίας: title, genre, and two cruces’, in D. Manetti (ed.), Studi sul De Indolentia di Galeno (Roma: Fabrizio Serra, 2012), 69–91: 77–9.

13. Stobaeus, *Anthology*, 2.7.2; cf. 2.39.20–41.25: *Ioannis Stobaei anthologium*, ed. Otto Hense and Curt Wachsmuth, (Berlin: Weidmann, 1884–1912). See Charles Brittain, *Philo of Larissa: The Last of the Academic Sceptics*(Oxford: Oxford University Press, 2001), 277–80. On the other hand, the genre of therapy of emotions in particular pre-dated Philo and goes back at least to Chrysippus’ ‘therapeutic’ Book 4 of his *On Passions*, see Gill, *Naturalistic Psychology*, *op. cit* (note 3), 280–300 and Teun Tieleman, *Chrysippus’ On Affections: Reconstruction and Interpretation* (Leiden: Brill, 2003), 140–97.

14. Mainly Boudon-Millot, ‘Un traité perdu’, *op. cit* (note 7), 75–6, who later reconsidered the generic identity of the essay, Boudon-Millot, Jouanna, Pietrobelli, *op. cit* (note 7), x. Traditional consolations include Cicero’s *Consolation* on the death of his daughter Tullia, Seneca’s *Consolation to Marcia* or *Consolation to Polybius*, Plutarch’s *Consolation to his wife*, and Pseudo-Plutarch’s *Consolation to Apollonius*

15. For the distinction between the categories of works on *alypia* and *paramythia*, Epictetus, *Discourses* 3.24.116, Heinrich Schenkl (ed.), *Epicteti dissertationes ab Arriano digestae*(Leipzig: Teubner, 1916).

16. The name of the grammarian is dubious. Vlatadon reads Philides; BM corrected to Philippides; Nutton in his English translation (cited notes 7, 79) suggests Philistides, but I follow the emendation of Kotzia in favour of Callistus, which is based on a close parallel to Galen’s commentary on *Hippocratic Epidemics VI*, Ernst Wenkebach and Franz Pfaff (eds), *Galeni in Hippocratis Sextum Librum Epidemiarum commentaria I-VI*(*CMG* V.10.2.2) (Berlin: in aedibus Academiae litterarum, 1956), 486, 19–24.

17. Book 2, Paul Kraus (ed.), ‘Kitâb al-Akhlâq li-Jâlînus’, *Bulletin of the Faculty of Arts of the Egyptian University* 5, 1 (1939), 1–51: 39; with English translation by John Mattock, ‘A translation of the Arabic epitome of Galen’s book *Peri êthôn*’, in S.M. Stern, A. Hourani, V. Brown (eds), *Islamic Philosophy and the Classical Tradition: Essays Presented by his Friends and Pupils to Richard Walzer on his Seventieth Birthday*(Oxford: Cassirer, 1972), 235–60: 248.

18. Michel Foucault, *The History of Sexuality*, vol. 3, *The Care of the Self*, trans. Robert Hurley) (London: Harmondsworth, 1990), 53. As Foucault stresses, works of the principate concerned with the interplay between the care of the Self and the help of the Other build on ‘pre-existing relations’ between author and reader and cause the ‘intensification of existing social relations’.

19. Mainly Susan Mattern, *Galen and the Rhetoric of Healing*(Baltimore, MD: John Hopkins University Press, 2008); Luis García Ballester, ‘Elementos para la construcción de las historias clínicas en Galeno’, *Dynamis*, 15 (1995), 47–65; cf. Cristina Álvarez-Millán, ‘Greco-Roman Case Histories and Their Influence on Islamic Clinical Accounts’, *Social History of Medicine*, 12 (1999), 19–43: 30–3; cf. Susan Mattern, ‘Galen’s Ideal Patient,’ *Asklepios*, 2 (2008), 116–30.

20. For *thaumazein* in case histories in relation to praise, see Mattern, *Galen and the Rhetoric of Healing*, *op. cit.*(note 19), 80–3. On the performative aspect of Galen’s anatomical demonstrations, see Maud Gleason, ‘Shock and awe: the performance dimension of Galen’s anatomy demonstrations’, in Gill, *et al.* (eds), *op. cit.* (note 2), 85–114.

21. *On Prognosis* 2, Kühn (ed.), *op. cit.*(note 1), vol. 14, 612 /article/back/fn-group/fn/p/mixed-citation/inline-formula  Nutton (ed.), *op. cit.* (note 1), *CMG*, vol. V.8.1, 80.22–25.

22. *On Prognosis* 11, Kühn (ed.), *op. cit.*(note 1), vol. 14, 657–660 /article/back/fn-group/fn/p/mixed-citation/inline-formula  Nutton (ed.), *op. cit.* (note 1), *CMG*, vol. V.8.1, 126.26–128.32.

23. *On Prognosis* 2, Kühn (ed.), *op. cit.*(note 1), vol. 14, 605 /article/back/fn-group/fn/p/mixed-citation/inline-formula  Nutton (ed.), *op. cit.* (note 1), *CMG*, vol. V.8.1, 74.15.

24. For the notion of ‘communality’ in Galen, see Jason König, ‘Self-promotion and self-effacement in Plutarch’s *Table Talk*’, in F. Klotz and K. Oikonomopoulou (eds), *The Philosopher’s Banquet: Plutarch’s Table Talk in the Intellectual Culture of the Roman Empire*(Oxford: Oxford University Press, 2011), 183–6. The term ‘sociative’ was coined by Friedrich Slotty, ‘Die Stellung des Griechischen und anderer idg. Sprachen zu dem soziativen und affektischen Gebrauch des Plurals der ersten Person’, *Indogermanische Forschungen*, 45 (1928), 348–63.

25. On *hypomnemata* in Plutarch, see Sophia Xenophontos, ‘Plutarch’s Compositional Technique in the *An Seni Respublica Gerenda Sit*: Clusters vs. Patterns’, *American Journal of Philology*, 113, 2 (2012), 61–91 with additional references.

26. Plutarch, ‘*De tranquillitate animi*’, in Max Pohlenz (ed.), *Plutarchi moralia*(Leipzig, Teubner, 1929; repr. 1972).

27. A.A.Long, Epictetus: A Stoic and Socratic Guide to Life (Oxford: Oxford University Press, 2002), 43–44 though of course this is often lost in the summaries in the *Manual* (*Encheiridion*). See also his pp. 52–66 on Epictetus’ styles of discourse.

28. On how suggestions can influence our cognitions and behaviours, see the new study by Robert Michael, Maryanne Garry and Irving Kirsch, ‘Suggestion, Cognition, and Behavior’, *Current Directions in Psychological Science*, 21, 3 (2012), 151–6 with additional references; also G. Colket Caner, ‘Use of suggestion in psychotherapy’, *The New England Journal of Medicine*, 251, 18 (1954), 733–5. For the practical application of suggestion in the medical sphere, including constructed statements that promote suggestion, see B.P.L., ‘Suggestions’, *The American Journal of Nursing*, 11.2 (1910), 126–7. For further reading, see Hippolyte Bernheim, *Die Suggestion und ihre Heilwirkung*(Tübingen: Edition diskord, 1888; repr. 1985), Boris Sidis, *The Psychology of Suggestion* (New York: Arno Press, 1973).

29. Ch. 2–4, Veronique Boudon (ed.), *Galien, Œuvres, Tome II: Exhortation à l’étude de la médecine: Art médical*(Paris: Belles Lettres, 2000), 85–8 /article/back/fn-group/fn/p/mixed-citation/inline-formula  Kühn (ed.), *op. cit* (note 1), vol. 1, 3–6.

30. Also in Galen’s *Exhortation to the Study of Medicine*, 5: see Boudon (ed.), *op. cit* (note 29), 90 /article/back/fn-group/fn/p/mixed-citation/inline-formula  Kühn (ed.), *op. cit* (note 1), vol. 1, 8–9.

31. For example, A.M.Wood, S. Joseph and S.P.A. Linley, ‘Gratitude: The Parent of all Virtues’, The Psychologist, 20 (2007), 18–21

32. Norman W. DeWitt, ‘The Epicurean Doctrine of Gratitude’, *Americal Journal of Philology*, 58, 3 (1937), 320–8. Cf. Voula Tsouna, ‘Epicurean therapeutic strategies’, in J. Warren (ed.), *The Cambridge Companion to Epicureanism*(Cambridge: Cambridge University Press, 2009), 249–65.

33. For Plato and Aristotle, ‘noble’ as a contrast to ‘malicious’ was one of the criteria for approval or disapproval of an ethical action: *Republic* 363e–364a: *Platonis Rempublicam*, ed. Simon Slings (Oxford: Clarendon Press, 2003); *Nicomachean Ethics* 1104b32: *Aristotelis ethica Nicomachea*, ed. Ingram Bywater (Oxford: Clarendon Press, 1894; repr. 1962).

34. Also in Diogenes Laertius, *Lives of Philosophers*7.5, *Diogenis Laertii vitae philosophorum*, ed. Herbert Strainge Long (Oxford: Clarendon Press, 1964; repr. 1966).

35. TadBrennan, ‘Stoic Moral Psychology’, in Brad Inwood (ed.), The Cambridge Companion to the Stoics (Cambridge: Cambridge University Press, 2003), 257–294: 269–74.

36. See Cicero’s formulation of ‘rerum externarum despicientia’ attributed to Panaetius in *On Duties* 1.66: *M. Tullius Cicero, De officiis*, ed. Walter Miller (Cambridge, MA: Harvard University Press, 1913).

37. Galen’s phrase ‘I knew nothing of these things on my first stay in Rome’ with reference to rivalries and vices [*On Prognosis* 2, Kühn (ed.), *op. cit.*(note 1), vol. 14, 605 /article/back/fn-group/fn/p/mixed-citation/inline-formula  Nutton (ed.), *op. cit.* (note 1), *CMG*, vol. V.8.1, 74, 12–13] suggests that he considered Pergamum a space of moral superiority. On the corruption of the medical profession, see for instance *On Recognising the Best Physician* 1: *Galeni de optimo medico cognoscendo libelli versio Arabica*, ed. Albert Iskandar (*CMG* Suppl. Or. IV) (Berlin: in aedibus Academiae litterarum, 1988), 41–7.

38. Seminal discussions include, Simon Swain, *Hellenism and Empire: Language, Classicism and Power in the Greek world, A.D. 50–250*(Oxford: Oxford University Press, 1996); Graham Anderson, *The Second Sophistic: A Cultural Phenomenon in the Roman Empire* (London: Routledge, 1993); Tim Whitmarsh, *Greek Literature and the Roman Empire: The Politics of Imitation* (Oxford: Oxford University Press, 2001), and S. Goldhill (ed.), *Being Greek under Rome: Cultural Identity, the Second Sophistic, and the Development of the Empire* (Cambridge: Cambridge University Press, 2001). On Galen’s complex relation to the ‘Second Sophistic’, see Heinrich von Staden, ‘Galen and the “Second Sophistic”’, *Bulletin of the Institute of Classical Studies*, 41 (1997), 33–54.

39. See, for instance, Mireille Armisen-Marchetti, ‘Imagination and meditation in Seneca: the example of praemediatio’, in J.G. Fitch (ed.), *Seneca: Oxford Readings in Classical Studies* (Oxford: Oxford University Press, 2008), 102–13. Studies on the techniques of ancient meditation include Paul Rabbow, *Seelenführung: Methodik der Exerzitien in der Antike*(Munich: Kösel, 1954), Ilsetraut Hadot, *Seneca und die griechisch-römische Tradition der Seelenleitung* (Berlin: Walter de Gruyter, 1969), and Pierre Hadot, *Exercices spirituels et philosophie antique* (Paris: Etudes augustiniennes, 1981). On the history of meditation in Imperial Stoicism, see R.J. Newman, ‘Cotidie meditare: Theory and Practice of the Meditatio in Imperial Stoicism’, *ANRW*, II.36.3 (1989), 1473–517.

40. MarcusAurelius, ‘Meditations, 7.33 and 7.64’, in Joachim Dalfen (ed.), Marci Aurelii Antonini ad se ipsum libri XII (Leipzig: Teubner, 1979).

41. From an unnamed Euripidean play; Theseus is the speaker: ‘as I once learned from a wise man,/article/back/fn-group/fn/p/mixed-citation/inline-formula  I fell to considering disasters constantly,/article/back/fn-group/fn/p/mixed-citation/inline-formula  imagining for myself exile from my native land,/article/back/fn-group/fn/p/mixed-citation/inline-formula and untimely deaths and many other misfortunes,/article/back/fn-group/fn/p/mixed-citation/inline-formula  so that if I ever suffer anything from what I was imagining /article/back/fn-group/fn/p/mixed-citation/inline-formula  it will not be unexpected and will not tear my soul apart’, fr. 964: *Tragicorum Graecorum Fragmenta*, ed. Richard Kannicht, vol. 5.2, (2004), (Göttingen: Vandenhoeck & Ruprecht, 1971–2004), 963. Cf. Galen, *On the Doctrines of Hippocrates and Plato* 4.7: *Galeni: De placitis Hippocratis et Platonis*, ed. Philip De Lacy, 3 vols (*CMG* V.4.1.2) (Berlin: in aedibus Academiae litterarum, 1978–84), 282, 17–23 /article/back/fn-group/fn/p/mixed-citation/inline-formula Kühn (ed.), *op. cit.* (note 1), vol. 5, 418.

42. For example, in his *Method of Healing*, 8.3, Kühn (ed.), *op. cit* (note 1), vol. 10, 560-1.

43. Book 1, Kraus (ed.), *op. cit* (note 17), 29; with Mattock, *op. cit.*(note 17), 239.

44. *Affections and Errors of the Soul* 7: *Galeni De propriorum animi cuiuslibet affectuum dignotione et curatione*; *De animi cuiuslibet peccatorum dignotione et curatione*; *De atra bile*, ed. Wilko De Boer, (Berlin: Teubner, 1937), 25, 20–1, *CMG*V.4.1.1 /article/back/fn-group/fn/p/mixed-citation/inline-formula Kühn (ed.), *op. cit* (note 1), vol. 5, 37.

45. 2: *Claudii Galeni Pergameni Scripta Minora*, vol. 3: Περί αἱρέσεων τοῖς εἰσαγομένοις, Θρασύβουλος, Περὶ φυσικῶν δυνάμεων ed. Georg Helmreich *et al.*(Leipzig: Teubner, 1893), 178–80.

46. 9.2: *op. cit.*, ed. De Lacy, (note 41), (*CMG* V.4.1.2) 550, 8–31 /article/back/fn-group/fn/p/mixed-citation/inline-formula Kühn (ed.), *op. cit.*(note 1), vol. 5, 732–4.

47. Note that in his medical work *The Best Method of Teaching*Galen supports the presence of a teacher supervisor, whose aim is to correct the mistakes that arise from the natural deficiencies of the student.

48. In the *Affections and Errors*Galen discusses an additional cause of distress next to material losses, the one that comes from inflicting one’s sense of shame upon one’s self, a feeling to which young people are especially prone. Again Galen plays with the social expectations of his addressees, as Graeco-Roman society was predominantly a society of *aidos*

49. An opinion developed by Cleanthes [Stobaeus, *Eclogues* 2.65.8-9: *Ioannis Stobaei Eclogarum physicarum et ethicarum libri duo*, ed. August Meineke (Leipzig: Teubner, 1860–4)], probably in his book *On Excellence of Natural Endowment*; (Diogenes Laertius, *Lives of the Philosophers* 7.175: *op. cit.*, ed. Long, (note 34), and by Chrysippus [Plutarch, *On Common Notions Against the Stoics*1069E: *Plutarchi moralia*, ed. Rolf Westman, vol. 6.2 (Leipzig: Teubner, 1959)].

50. 8: *op. cit.*, ed. De Boer, (note 44), *CMG*, vol. V.4.1.1, 27.22–28.7 /article/back/fn-group/fn/p/mixed-citation/inline-formula  Kühn (ed.), *op. cit* (note 1), vol. 5, 40–1.

51. 8: *op. cit.*, ed. De Boer, (note 44), *CMG*, vol. V.4.1.1, 30.10–16 /article/back/fn-group/fn/p/mixed-citation/inline-formula  Kühn (ed.), *op. cit* (note 1), vol. 5, 44–5.

52. *Method of Healing*, 7.1, Kühn (ed.), *op. cit* (note 1), vol. 10, 457.

53. Galen readjusts his emphases to the level of his audience very frequently, eg. ‘I have also called the faculties within this soul ‘nature’ in my book *On the Natural Faculties*, because in this tract I was dealing with ordinary doctors’; *On My Own Opinions*, 3, 19–23; Veronique Boudon-Millot and Antoine Pietrobelli (eds), ‘Galien ressuscité: Édition princeps du texte grec du *De propriis placitis*’, *Revue des études grecques* 118, (2005), 168–213: 174.

54. Cicero, *Tuscalan Disputations* II.17-8, V.31: *Tusculanea Disputationes*, ed. Max Pohlenz (Leipzig: Teubner 1918); Plutarch, *That it is Impossible to live a Pleasant Life according to Epicurus* 1088B, 1090A: *op. cit.*, ed. Rolf Westman, (note 49). The same was the case with the Stoic sage, who was expected to have risen above the emotions of pain or anger, *Stoicorum Veterum Fragmenta* 3.586, ed. Hans Freidrich von Arnim (Stuttgart: Teubner, 1903–24; repr. 1978–9).

55. Gill, *Naturalistic Psychology*, *op. cit* (note 3), 259–60; Hankinson, (1993) ‘Actions and Passions’, *op. cit* (note 3): 198–204, Donini, *op. cit.*(note 3): 194; Singer, *Psychological Writings*, *op. cit* (note 7), 22.

56. Cf. for instance, Lieve van Hoof, *Plutarch’s Practical Ethics: The Social Dynamics of Philosophy*(Oxford: Oxford University Press, 2010), 160–1.

57. Cf. ch. 4–5, *Exhortation to the Study of Medicine*, Boudon (ed.), *op. cit.*(note 29), 87–9 /article/back/fn-group/fn/p/mixed-citation/inline-formula  Kühn (ed.), *op. cit* (note 1), vol. 1, 5–7, for groups of people with whom Galen discourages identification.

58. 1.1.7: *Galeni De alimentorum facultatibus libri III*, *De bonis malisque sucis*(*CMG* V.4,2), ed. Georg Helmreich (Berlin: Teubner, 1923) 204, 3–5 /article/back/fn-group/fn/p/mixed-citation/inline-formula  Kühn (ed.), *op. cit.* (note 1), vol. 6, 457.

59. Philip van der Eijk, ‘Galen’s use of the concept of “qualified experience” in his dietetic and pharmacological works’, in A. Debru (ed.), *Galen on Pharmacology: Philosophy, History and Medicine*(Leiden: Brill, 1997), 35–58: Laurence Totelin, ‘And to end on a poetic note: Galen’s authorial strategies in the pharmacological books’, *Studies in History and Philosophy of Science Part A*, 43, 2 (2012), 307–15. In Galen’s technical texts, *peira* (contrasted with mere *logos*) is usually connected with his self-promotion strategy and the construction of his authority, see Vivian Nutton, ‘Galen’s authorial voice: a preliminary enquiry’, in L. Taub and A. Doody (eds), *Authorial Voices in Greco-Roman Technical Writing* (Trier: Wissenschaftlicher Verlag, 2009), 53–62. See also, Heinrich von Staden, ‘Author and authority: Celsus and the construction of a scientific self’, in M.E. Vázquez Buján (ed.), *Tradición e innovación de la medicina latina de la antigüedad y de la Alta Edad Media* (Santiago de Compostela: Universidade de Santiago de Compostela, 1994), 103–17.

60. Cf. the distinction between ‘core’ and ‘extra’ in Singer, *Psychological writings*, *op. cit* (note 7), 11–12; also the label of ‘smaller-scale, more “occasional” type of literary production’ with reference to Galen’s psychological writings on p.2.

61. Singer, *Psychological Writings*, *op. cit* (note 7), 7–8.

62. An allusion to the common Byzantine perception of Galen and other doctors as longing for money.

63. Preferring βίῳ instead of the manuscript reading βίᾳ.

64. 81 KS /article/back/fn-group/fn/p/mixed-citation/inline-formula  26 BJP.

